# A pragmatic multi-setting lifestyle intervention to improve leisure-time physical activity from adolescence to young adulthood: the vital role of sex and intervention onset time

**DOI:** 10.1186/s12966-022-01301-4

**Published:** 2022-06-08

**Authors:** Parnian Parvin, Parisa Amiri, Hasti Masihay-Akbar, Mahnaz Khalafehnilsaz, Leila Cheraghi, Arash Ghanbarian, Fereidoun Azizi

**Affiliations:** 1 Research Center for Social Determinants of Health, Research Institute for Endocrine Sciences, Shahid Beheshti University of Medical Sciences, Tehran, Iran; 2 Academic Center for Education, Culture and Research, Shahid Behehsti University of Medical Sciences, Tehran, Iran; 3 Department of Epidemiology and Biostatistics, Research Institute for Endocrine Sciences, Shahid Beheshti University of Medical Sciences, Tehran, Iran; 4 Prevention of Metabolic Disorders Research Center, Research Institute for Endocrine Sciences, Shahid Beheshti University of Medical Sciences, Tehran, Iran; 5 Endocrine Research Center, Research Institute for Endocrine Sciences, Shahid Beheshti University of Medical Sciences, Tehran, Iran

**Keywords:** Healthy lifestyle education, Physical activity, Adolescents

## Abstract

**Background:**

The long-term effectiveness of healthy lifestyle interventions on improving leisure-time physical activity (LTPA) in adolescents and its factors in low- and middle-income communities is unclear. This study is the first to investigate LTPA trends in a population of Iranian adolescents who underwent a multi-setting lifestyle intervention, considering sex and the time of intervention onset.

**Methods:**

Participants were 2374 adolescents (57.2% girls), aged 12–18 years, who participated in the Tehran Lipid and Glucose Study (TLGS) during 1999–2001 and followed for a median follow-up of 15.9 over five data points every 3 years. Adolescent participants were assigned to the intervention or control groups based on their residential areas. Boys and girls were categorized into 12–15 or 16–18 years old to minimize participants’ bio-psychological differences, facilitate environmental interventions by more compliance with the Iranian educational system and identify the best time to start the intervention. All adolescents in the intervention area received healthy lifestyle interventions via the school-, family-, and community-based programs. LTPA was assessed using the reliable and validated Iranian Modifiable Activity Questionnaire (MAQ) version over the five data points. The Generalized Estimating Equations method was used to evaluate educational intervention’s effect on LTPA in adolescents during the follow-up.

**Results:**

In boys who experienced the early onset of intervention (12–15 years), the interaction effect of follow-up examinations and the intervention was significant where the impact of the intervention differed over time. In this group, LTPA was higher in the control group than in the intervention group at the first follow-up examination (β = − 1088.54). However, an increasing trend of LTPA was observed in the intervention group until the third follow-up examination (β = 1278.21, *p* = 0.08, and β = 1962.81, *p* = 0.02, respectively), with borderline significance levels at the 2nd (*P* = 0.08) and the 4th (P = 0.08) measurements. The interaction terms and main effects of intervention and follow-up examinations were not significant in boys with late intervention onset. Although older boys in the intervention group had higher LTPA than the control group, there were no significant differences among study groups in all follow-up examinations. Regarding girls, LTPA did not differ significantly between intervention and control groups in all follow-up examinations (*P* > 0.05).

**Conclusion:**

Our results showed that a multi-setting practical lifestyle intervention could improve long-term energy expenditure in LTPA in adolescent boys who have experienced an early onset intervention. Findings emphasized the vital role of gender and the onset of these interventions. The current results would be valuable to plan tailored interventions to improve LTPA and community health.

**Trial registration:**

This study is registered at Iran Registry for Clinical Trials (IRCT), a WHO primary registry (http://irct.ir). The TLGS clinical trial was the very first registration in the IRCT (Iran Registry of Clinical Trials). it was registered on 2008-10-29 by the registration number IRCT138705301058N1. Based on the international committee of medical journal Editors (ICMJE), “retrospective registration” is acceptable for trials that began before July 1, 2005.

## Background

The decreasing trend of physical activity is a global problem [1]. Worldwide, one in four adults, and three in four adolescents aged 11–17 years, do not currently meet the international recommendations for physical activity [2]. National studies showed that about 40% of Iranian adults and more than 23% of Iranian children and adolescents do not have enough daily physical activity [3, 4]. Also, the study of the National Surveillance of Risk Factors of Non-Communicable Diseases (SuRFNCD) conducted from 2006 to 2011 in Iran showed an increasing trend of low physical activity in urban and rural adolescents. Urban girls, meanwhile, were the most vulnerable group [5].

Leisure-time physical activity (LTPA) refers to the preferred physical activities done in individuals’ free time [6]. Although LTPA is most effective in various aspects of human health [7], the available evidence suggests that studies specifically pay insufficient attention to this dimension in related interventions. One of the most important factors neglected is the timing of the start of these interventions. It is well documented that modifying unhealthy behaviors is more complicated by transitioning from early to late adolescence [8]. Therefore, the onset time of behavioral interventions in adolescents should be considered essential in relevant planning. In addition, previous studies have shown significant sex differences in accepting healthy behaviors [9]. Evidence confirms that behavioral interventions to promote physical activity significantly impact younger participants and males [2, 10]. Various factors, including physiologic growth and development process and the need to accept new social roles, could be the most important reasons for this sex/age-specific trend in physical activity [11, 12]. The mentioned sex differences would be even more dominant in Eastern countries, especially Muslim societies, where socio-cultural values and limited access to facilities are the main barriers for females’ physical activity [13, 14]. Despite mentioned evidence, most physical activity trials neither stratified early and late adolescents nor reported gender-specific results. Hence, there is still a need to address these critical aspects in developing and implementing relevant interventions.

Further to the mentioned gaps, the best settings to achieve sustainable improvement in adolescents’ physical activity are debated. A systematic review of reviews in 2019 indicated that multi-component school-based interventions with parental involvement might be the most effective plan to improve physical activity [15]. However, a more recent review article has questioned this finding [16]. The last review focused on the age range of six to18 years, and again the impact of the onset time of interventions, especially in the early and late adolescence, is not clear. On the other hand, most of the interventions in this field have focused on short-term changes in physical activity, and the process of these changes in the long term needs further investigation [15].

In Iran, few randomized trials have examined the short-term impact of behavioral interventions on students’ physical activity. Overall, the results showed improved physical activity after the interventions [17, 18]. However, there is still no reliable information about the effects of early practical interventions in the community to promote LTPA from an early age. Iran is among the countries that designed and implemented a national primary healthcare-based program entitled the IRAN-Ending Childhood Obesity (IRAN-ECHO) in the framework of the WHO-ECHO program from 2015. Promoting adolescents’ physical activity at the individual and community levels is one of the critical strategies of the IRAN-ECHO program. There is still a need to gather the necessary information in advancing such health promotion plans and their practical implementation at the national level. Tehran Lipid and Glucose Study (TLGS) is a long-term multi-setting lifestyle intervention that has been implemented in schools, families, and the community. The study’s main goal was to promote healthy lifestyles via improving nutrition and dietary patterns, increasing physical activity levels, and smoking cessation, leading to primary prevention of cardio-metabolic risk factors and NCDs. It was adopted from successful large-scale interventions like the American Heart Association guidelines and the North Karelia project [19, 20]. In the framework of the TLGS, the current study, for the first time, aimed to investigate the long-term effects of early and late-onset of the mentioned lifestyle intervention on LTPA levels in girls and boys and related energy expenditure. In addition, to respond to existing worldwide gaps, the present results can provide valuable evidence for related interventions in other Middle-Eastern countries with similar cultural, social, and religious contexts.

## Methods

### Study design and participants

The current study was conducted within the Tehran Lipid and Glucose Study (TLGS) framework, a population-based cohort study to determine the risk factors for non-communicable diseases (NCDs) among an urban population of Tehran. The study’s main objectives included: 1) Determining the prevalence of NCDs and their associated risk factors through a cross-sectional phase (1999–2001) followed by five follow-up examinations at 3-year intervals (2002–2004, 2005–2007, 2008–2010, 2011–2013, 2014–2016); 2) Investigating the effects of a healthy lifestyle intervention on NCD risk factors and outcomes.

The multistage cluster random sampling method was used to select the target sample. Of 20 health care centers located in district 13 of Tehran, three centers with complete information about the families covered were selected. In the first phase of the TLGS, participants were randomly selected from the population covered by the mentioned health care centers. As a field trial, in the second phase of the TLGS, participants under the coverage of one of the health care centers were considered an intervention group. Of the total 7151 families (intervention area: 2237, control areas: 2056 and 2858), 4751 families (intervention area: 1816, control areas: 1261 and 1674) were randomly selected to participate in the study. Thus, in total, 15,005 individuals (aged ≥ three years old) from randomly selected families participated in the baseline measurement of the TLGS. Of those, 5630 participants received the lifestyle intervention. The assignment of participants to the intervention and control groups was based on their area of residency. Far from the other two, one of the health care centers was selected as the intervention center, and the individuals residing in the area under its coverage received the intervention. Those living in areas covered by two other health care centers were selected as the control group and received routine and nationally approved health care [21, 22].

**Fig. 1:**
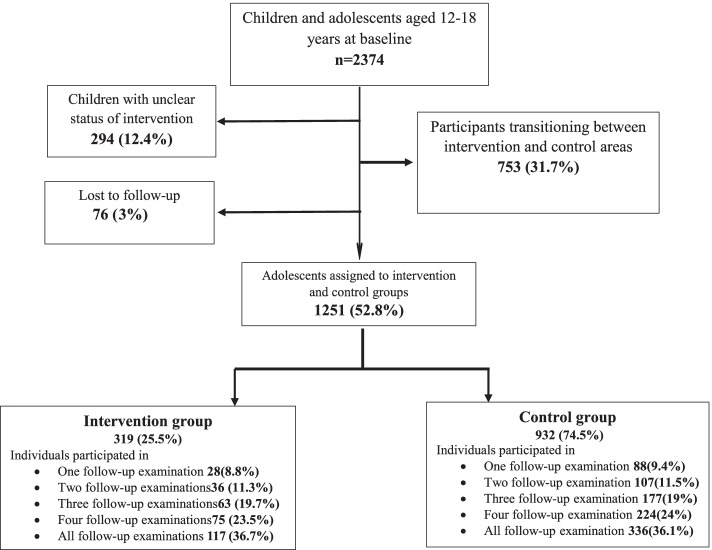
The sampling flowchart

In the current analysis, data on 2374 adolescents (57.2% girls), aged 12–18 years, who participated in the baseline assessment of the TLGS were considered and monitored for over a median follow-up of 17.0 years. Of those recruited for the study, 1123 participants were excluded for the following reasons: lack of residential address at the time of data gathering (294, 12.4%), those who were displaced between study groups (753, 31.7%), and lost to follow up in all examinations (76, 3%). Final analysis has been conducted on 1251 eligible adolescents assigned to two groups control (932, 74.5%) and intervention (319, 25.5%). Further details of the current sampling design are illustrated in Fig. 1.

### The outcome variable

In the current study, energy expenditure, defined as the calories an individual will burn during LTPA, was considered a continuous dependent variable from adolescence to adulthood. The LTPA data was collected using the validated Iranian version of the Modifiable Activity Questionnaire (MAQ) in all follow-up examinations [23]. In this regard, the adolescents’ version of the questionnaire [23] was used for participants aged 12–18 years, and an adult version [24] was used for those aged > 18 years old during follow-up. Both versions include 15 Iranian popular and common activities specified for the mentioned age groups during leisure time and time spent in each activity. To calculate the energy expenditure in LTPA, we multiplied the total weekly exercise minutes dedicated to each activity (Time spent in LTPA) by its typical intensity expressed in metabolic equivalents (METs) and individual’s weight to obtain METs.Kg- min/wk. [25]. Time spent in LTPA was estimated by the number of months a year and time per week that every activity was performed, considering possible seasonal variation.

Metabolic equivalent task (MET) is a physiological measure expressing the energy cost of physical activity. It is defined as the ratio of metabolic rate (or energy consumption) during a specific physical activity to a reference metabolic rate, set by convention to 3.5 ml O2 kg-1•min-1. In many studies, MET-value is defined as the energy expenditure of a particular physical activity divided by per kg body weight and hour and calculated by 1 MET = 1 kcal / (Kg*h). In other words, regardless of duration, MET-values are assigned to a particular activity to describe its intensity [26].

Moreover, baseline physical activity in the TLGS was assessed using the Lipid Research Clinics (LRC) questionnaire, which evaluated this behavior based on three sub-scales: regular, strenuous, and self-rated physical activity. Results have been classified as high (at least three times a week), moderate (less than three times a week), and low (none in the past week) [27].

### Study measurements

Participants’ BMI [weight (kg)/height (m2)] was calculated based on weight and height values in each follow-up examination. Weight was measured, with subjects minimally clothed without shoes, using digital scales (Seca 707: range 0–150 kg) and recorded to the nearest 1 kg. Using a tape meter, height was measured in a standing position without shoes, while shoulders were in normal alignment. To determine body weight status in children aged ≤19 years, WHO percentiles for body mass index (BMI)-for-age and cutoffs for bodyweight status were used [28], and BMI between 25 to 30 kg/m2 and BMI ≥ 30 kg/m2 were defined as overweight and obesity cutoff points in adults.

Level of education was categorized based on the participants’ total years of education; 0–5 years as “primary”, 6–12 years as “secondary”, and over 12 years as “higher” (including university courses). Employment status was categorized depending on whether the participant has a job or not.

### Multi-setting intervention

Face-to-face counseling, written materials, public health education, and the promotion of health policies to facilitate healthy lifestyles among the intervention group were strategies used at the family, school, and community levels. The TLGS scientific committee prepared the intervention content in each context delivered under the supervision of the public health care center located in the intervention area.

#### Schools

Twelve schools in the intervention area farthest from the control area were selected as “Health Promoting Schools”. These schools implemented the school-based subprogram, which focused on improving children’s physical activity and healthy eating as well as reducing smoking. The scientific committee trained the principals and volunteer teachers regarding a healthy lifestyle, taking students’ age and needs into account, at the beginning of each year. All students in the first year of both guidance (12–15 years) and high schools (16–18 years) participated in nine 45-minute classes. Volunteer students formed a “school health society” under the supervision of their teachers and transferred health messages to their peers. In addition, family involvement was an essential part of the school-based program. Parents were educated to form a supportive and healthy environment at home. Recurrent parent-teacher meetings and annual seminars were held to maintain families’ engagement. Also, pamphlets/booklets with healthy lifestyle-related content were distributed between families. The number and duration of educational sessions and the number of participants (students and parents) were registered based on the study protocol. Almost 70% of the planned school-based interventions were successfully implemented.

#### Families

Parents were involved in forming a supportive and healthy home environment. The family involvement component aimed to introduce parents to the school-based lifestyle modification program and assist them in creating a supportive environment to improve healthy behaviors in adolescents, including LTPA. Through ordinary meetings of the parent-teachers in each school, interactive forums were conducted to inform parents regarding alarming rates of NCD risk factors in Iran, the necessity of lifestyle modification, and relevant practical recommendations.

In addition, family-based interventions were delivered by inviting families for group sessions (for > 2 hours, 10–12 individuals in each session) between baseline and the first follow-up examination. Families were also involved in the lifestyle modification program by “Health liaisons”, who were volunteers responsible for delivering the program under the supervision of the public health care center in the intervention area. All families in the intervention area received pamphlets/booklets containing information and benefits of healthy lifestyle behaviors (physical activity, food pyramid, smoking consequences, cessation tips, and coping with stress). They also received a seasonal newsletter named “Courier of Health,” containing affordable recommendations regarding healthy lifestyle behaviors. To sensitize and motivate families regarding lifestyle change, some results of the TLGS were reported in the mentioned health newsletter (mainly about the prevalence and the effect of risk factors in their area). Telephone surveys showed that 50% of households had received and paid attention to educational pamphlets and health newsletters.

#### Community

Community-based components of the TLGS intervention engaged various sectors, i.e., municipality, police, media, and community and religious leaders. Two to four times a year, on religious occasions such as Ramadan and special days such as World No Tobacco Day or World Diabetes Day, ceremonies were held at the local mosque or amphitheater. In these ceremonies, health messages were conveyed to the public through lectures on healthy living. In addition, city billboards in the intervention area were used for advertising a healthy lifestyle. More than 80% of the households participated in at least one public gathering between every two examinations.

### Statistical analysis

Mean ± SD and frequency (percent) were reported as data descriptions for continuous and categorical variables, respectively. The Chi-square test and independent samples T-test were conducted for group comparisons. Generalized Estimating Equations (GEE) as a robust statistical method was used to evaluate educational intervention’s effect on leisure-time physical activity in adolescents during 15.9 years of follow-up. The GEE procedure considered the “identity” link function and “autoregressive” working correlation matrix. The main effects of follow-up times and intervention and their interaction terms on physical activity were examined. GEE models were adjusted for age, education, and occupation at each follow-up and BMI at baseline. In the present study, parental characteristics that significantly differed in the intervention and control groups were explicitly adjusted in each age- and sex-specific group. Hence, parental ages and paternal occupation for younger girls and maternal education for older boys were considered adjusting factors Regarding parental characteristics, those factors that were significantly different between the control and intervention groups were adjusted considering adolescents’ sex and age. Parental ages and paternal occupation for younger girls and maternal education for older boys were considered adjusting factors. Adolescents’ and parental characteristics were compared between responders and non-respondents participants. All analyzes were performed based on gender and age (early and late adolescents). IBM SPSS Statistics 23 was used for statistical analysis.

## Results

Table 1 displays the distribution of participants’ BMI status and parental baseline characteristics in both intervention and control groups, considering adolescents’ age and gender. Except for BMI in younger adolescent girls (24.5% versus 12.9% overweight in control and intervention groups, respectively, *P* = 0.02), there were no significant differences between control and intervention regarding adolescents’ measured variables (*P* > 0.05). No parental variable was significantly different between control and intervention groups in early adolescent boys and late-adolescent girls. Parental age and fathers’ employment differed in late adolescent girls between intervention and control groups. This was only the case for maternal education levels in early adolescent boys.

**Table 1:** Baseline characteristics of the study participants (1999–2001)

	Early adolescence (12–15 years)						Late adolescence (16–18 years)					
	Boys			Girls			Boys		Girls			
	Control ***n*** = 273	Intervention ***n*** = 85	P	Control ***n*** = 293	Intervention ***n*** = 96	P	Control ***n*** = 153	Intervention ***n*** = 59	P	Control ***n*** = 213	Intervention ***n*** = 79	P
**Children characteristics**												
**BMI Status**			0.66			**0.02**			0.72			0.61
Normal	209 (77.4)	68 (80.0)		216 (75.5)	81 (87.1)		117 (77.5)	44 (74.6)		166 (79.8)	64 (83.1)	
Overweight	61 (22.6)	17 (20.0)		70 (24.5)	12 (12.9)		34 (22.6)	15 (25.4)		42 (20.2)	13 (16.9)	
**Physical activity**			0.57			0.37			0.09			0.82
Low	12 (10.4)	6 (15.8)		58 (37.2)	12 (22.6)		47 (32.0)	19 (32.8)		63 (29.9)	20 (26.3)	
Moderate	36 (31.2)	13 (34.2)		53 (34.0)	26 (49.1)		36 (24.5)	17 (29.3)		53 (25.1)	21 (27.6)	
High	67 (58.3)	19 (50.0)		45 (28.8)	15 (28.3)		64 (43.5)	22 (37.9)		95 (45.0)	35 (46.1)	
**Fathers’ characteristics**												
**Age**	48.8 ± 8.1	49.4 ± 7.5	0.57	47.3 ± 7.4	50.3 ± 9.0	**0.007**	50.2 ± 7.9	50.9 ± 7.7	0.58	51.4 ± 7.7	51.6 ± 7.9	0.83
**Physical activity**			0.32			0.15			0.23			0.62
Low	153 (59.3)	53 (63.1)		166 (61.5)	66 (71.7)		83 (58.0)	29 (53.7)		117 (61.9)	47 (66.2)	
Moderate	24 (9.3)	11 (13.1)		33 (12.2)	6 (6.5)		18 (12.6)	12 (22.2)		21 (11.1)	9 (12.7)	
High	81 (31.4)	20 (23.8)		71 (26.3)	20 (21.7)		42 (29.4)	13 (24.1)		51 (27.0)	15 (21.1)	
**BMI Status**			0.68			0.28			0.23			0.25
Normal	76 (33.9)	28 (37.3)		90 (39.6)	26 (32.1)		43 (31.6)	21 (41.2)		51 (34.0)	15 (25.4)	
Overweight	148 (66.1)	47 (62.7)		137 (60.4)	55 (67.9)		93 (68.4)	30 (58.8)		99 (66.0)	44 (74.6)	
**Education level**			0.23			0.56			0.41			0.24
Primary	113 (449)	45 (60.0)		103 (45.3)	44 (53.7)		69 (50.4)	29 (55.8)		69 (50.4)	29 (55.8)	
Secondary	77 (33.9)	18 (24.0)		86 (37.7)	27 (32.9)		48 (35.0)	14 (26.9)		48 (35.0)	14 (26.9)	
Higher	37 (16.3)	12 (16.0)		39 (17.1)	11 (13.4)		20 (14.6)	9 (17.3)		20 (14.6)	9 (17.3)	
**Employment status**			0.73			**0.005**			1.00			0.14
Employed	187 (82.4)	61 (80.3)		205 (89.5)	63 (75.9)		104 (75.4)	40 (76.9)		123 (80.9)	43 (71.7)	
**Mothers’ characteristics**												
**Age**	41.6 ± 6.8	49.4 ± 7.5	0.18	40.4 ± 6.6	43.5 ± 7.7	**< 0.001**	43.0 ± 6.8	44.0 ± 6.2	0.34	44.1 ± 6.8	43.6 ± 6.6	0.58
**Physical activity**			0.98			0.26			0.67			0.65
Low	137 (62.6)	43 (63.2)		126 (57.5)	41 (53.9)		80 (64.5)	28 (59.6)		85 (62.0)	36 (69.2)	
Moderate	28 (12.8)	8 (11.8)		43 (19.6)	11 (14.5)		17 (13.7)	9 (19.1)		16 (11.7)	5 (9.6)	
High	54 (24.7)	17 (25.0)		50 (22.8)	24 (31.6)		27 (21.8)	10 (21.3)		36 (26.3)	11 (21.2)	
**BMI Status**			0.45			0.24			0.84			0.477
Normal	48 (18.4)	21 (22.6)		53 (19.1)	24 (24.7)		24 (15.5)	10 (16.9)		32 (15.8)	15 (19.5)	
Overweight	213 (81.6)	72 (77.4)		225 (80.9)	73 (75.3)		131 (84.5)	49 (83.1)		170 (84.2)	62 (80.5)	
**Education level**			0.71			0.06			**0.03**			0.70
Primary	172 (64.4)	65 (69.1)		163 (57.6)	70 (70.0)		163 (57.6)	108 (68.4)		155 (75.2)	61 (79.2)	
Secondary	88 (33.0)	27 (28.7)		100 (35.3)	26 (26.0)		100 (35.3)	46 (29.1)		42 (20.4)	14 (18.2)	
Higher	7 (2.6)	2 (2.1)		20 (7.1)	4 (4.0)		20 (7.1)	4 (2.5)		9 (4.4)	2 (2.6)	
**Employment status**			0.40			0.52			0.80			0.56
Employed	26 (9.7)	6 (6.4)		25 (8.8)	6 (6.0)		16 (10.1)	7 (11.9)		10 (9.4)	5 (6.5)	

**Fig. 2:**
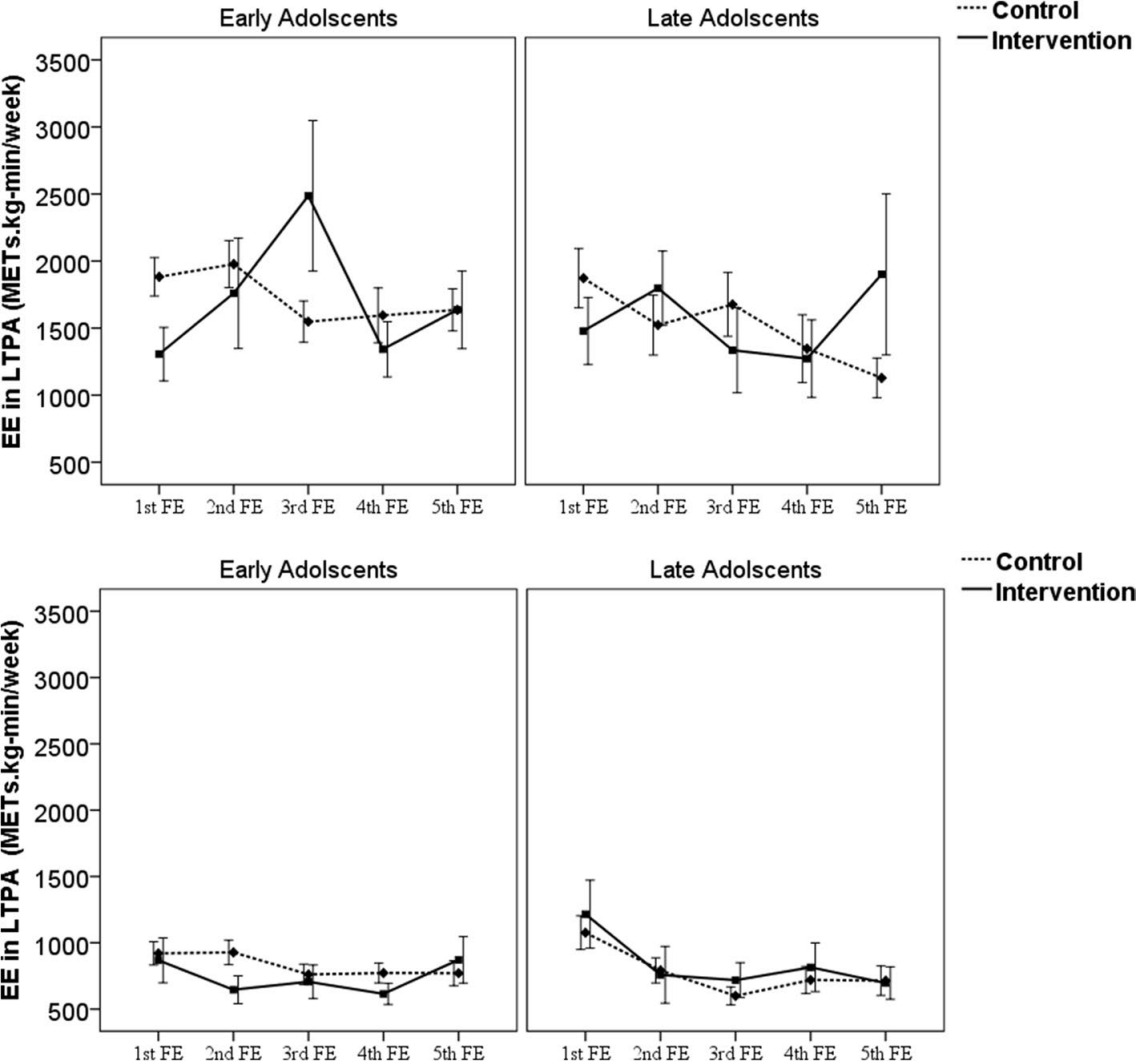
Mean of leisure-time physical activity in two study groups during five follow-up examinations. The median years after baseline assessment for each follow-up examination (FE): 1st FE: 3.6, 2nd FE: 6.7, 3rd FE: 10.4, 4th FE: 13.7, 5th FE:17.0. Baseline LTPA was assessed using the Lipid Research Clinics questionnaire (LRC). Corresponding values have been added to Table 1 in each study groups

The trend of LTPA in the intervention and control groups by sex is illustrated in Fig. 2. Higher LTPA levels were observed in boys than girls in both age groups in all follow-up examinations. Sex- and age-specific trends of leisure-time physical activity were observed in both intervention and control groups during follow-up examinations. In girls, this difference between control and intervention groups is less noticeable, and even in their late adolescence, both control and intervention groups show a declining trend in physical activity.

Using GEE models, the effectiveness of the intervention on individuals’ LTPA was separately evaluated in both sex- and age groups during follow-up examinations. Parental characteristics that significantly differed in the intervention and control groups were explicitly adjusted in each age- and sex-specific group. Hence, parental ages and paternal occupation for younger girls and maternal education for older boys were considered adjusting factors. The interaction effect of intervention with the follow-up examination time was also included in the model for examining the potential difference in physical activity changes in study groups (Table 2). In younger boys, the interaction effect of follow-up time with the intervention was significant, meaning that the impact of the intervention differed over time. Adjusted results showed that although in younger boys at the first follow-up, physical activity levels in the control group were higher than in the intervention group (β = − 1088.538), an increasing trend in favor of the intervention group was observed until the last follow-up, which was significant in the 3rd and 5th examinations (βs for interaction was respectively as 1962.81, and 1284.32). However, the interaction terms and main effects of intervention and follow-up examination were not substantial in older boys and girls.

**Table 2:** Adjusted effect of the intervention on leisure-time physical activity among adolescents estimated by GEE

		Boys		Girls	
		β (95% CI)	***P***-value	β (95% CI)	***P***-value
**Early adolescence**					
**Group**					
Intervention		−1088.54 (− 1798.15, − 378.93)	0.003	−19.30 (−798.07, 759.47)	0.96
**Time**					
2nd Follow-up		−31.31 (− 889.48, 826.26)	0.94	−40.01 (− 490.77, 410.76)	0.86
3rd Follow-up		− 624.79 (− 1803.65, 544.07)	0.30	− 183.51 (− 823.51, 456.48)	0.57
4th Follow-up		− 712.87 (− 2258.09, 832.35)	0.37	−164.87 (− 986.65, 656.90)	0.69
5th Follow-up		− 1135.76 (− 3227.50, 955.97)	0.29	208.11 (− 916.58, 1333.08)	0.72
Interaction Group*Time					
Interaction (F2 × Int)		1278.21 (− 169.97, 2726.39)	0.08	− 312.42 (− 1151.85, 527.02)	0.47
Interaction (F3 × Int)		1962.81 (306.62, 3619.01)	**0.02**	− 100.04 (− 1034.05, 833.97)	0.83
Interaction (F4 × Int)		1067.07 (−126.44, 2260.57)	0.08	−12.70 (− 878.53, 853.12)	0.98
Interaction (F5 × Int)		1284.32 (339.28, 2229.37)	**0.008**	234.49 (− 877.24, 3103.04)	0.68
**Late adolescence**					
**Group**					
Intervention		− 303.96 (− 1485.97, 878.05)	0.61	− 494.77 (− 1112.76, 123.21)	0.12
**Time**					
2nd Follow-up		− 596.12 (− 1617.44, 425.19)	0.25	−202.85 (− 704.78, 299.09)	0.49
3rd Follow-up		− 781.58(− 2201.33, 638.17)	0.28	− 147.63(− 767.64, 472.37)	0.64
4th Follow-up		− 1241.43 (− 3040.69, 611.83)	0.19	269.62 (− 678.97, 1218.21)	0.58
5th Follow-up		− 1210.47 (− 3526.63, 1141.69)	0.31	719.55 (−611.80, 2050.89)	0.29
Interaction Group*Time					
Interaction (F2 × Int)		1046.97 (− 298.22, 2392.17)	0.13	615.36 (− 238.90, 1469.62)	0.16
Interaction (F3 × Int)		371.45 (− 1146.61, 1889.50)	0.63	640.59 (−96.80, 1377.98)	0.09
Interaction (F4 × Int)		846.06(−647.84, 2339.96)	0.27	554.79 (− 429.72, 1539.31)	0.27
Interaction (F5 × Int)		1739.88 (−1135.36, 4615.12)	0.24	−61.67 (− 898.26, 774.92)	0.23

The 2nd, 3rd, 4th, and 5th follow-ups refer to the median time as 6.7, 10.4, 13.7, and 17.0 years after baseline assessment (1999–2001). The control group and the first follow-up examination (3.6 median years after baseline assessment) were considered reference categories.

GEE models were adjusted for age, education, and occupation at each follow-up and BMI at baseline. For younger girls, parental ages, paternal employment, and maternal education were also considered adjustments for older boys.

*Int* Intervention, *F* Follow-up, *CI* Confidence Interval, *β* regression coefficients that represent the mean differences of LTPA compared to the reference category.

Nonrespondent analysis results are presented in Table 3. Except for paternal weight (*P* = 0.002) and educational levels (*P* = 0.04), remaining socio-demographic and behavioral characteristics in adolescents and parents were not significantly different between respondent and non-respondent groups at baseline examination.

**Table 3:** Comparison of individual and parental baseline characteristics among respondent and non-respondent participants

	Non- responders^a^ (***n*** = 76)	Responders (***n*** = 1251)	***P***-value
**Children characteristic**			
**Age**	15.43 ± 2.19	14.98 ± 1.94	0.47
**Sex**			0.81
Boy	36 (47.4)	573 (45.8)	
Girl	40 (52.6)	678 (54.2)	
**Physical activity**			0.10
Low	25 (46.3)	287 (33.6)	
Moderate	10 (18.5)	255 (29.9)	
High	19 (35.2)	312 (36.5)	
**BMI Status**	20.68 ± 4.97	20.78 ± 4.16	0.84
**Fathers’ characteristics**			
**Age**	50.46 ± 6.50	49.43 ± 8.04	0.36
**Physical activity**			0.07
Low	38 (73.1)	580 (61.2)	
Moderate	2 (3.8)	138 (14.6)	
High	12 (23.1)	130 (24.3)	
**BMI Status**			0.002
Normal	8 (15.1)	342 (36.0)	
Overweight	45 (84.9)	608 (64.0)	
**Education level**			0.04
Primary	28 (51.9)	347 (36.2)	
Secondary	22 (40.7)	465 (48.5)	
Higher	4 (7.4)	146 (15.2)	
**Employment status**			0.21
Unemployed	14 (25.9)	177 (18.4)	
Employed	40 (74.1)	786 (81.6)	
**Mothers’ characteristics**			
**Age**	42.18 ± 6.69	42.39 ± 6.90	0.81
**Physical activity**			0.24
Low	47 (71.2)	716 (61.4)	
Moderate	7 (10.6)	136 (11.7)	
High	12 (18.2)	315 (27.0)	
**BMI Status**			0.87
Normal	11 (16.9)	216 (18.7)	
Overweight	54 (83.1)	941 (81.3)	
**Education level**			0.35
Primary	34 (51.5)	502 (46.6)	
Secondary	30 (45.5)	625 (53.1)	
Higher	2 (3.0)	51 (4.3)	
**Employment status**			0.36
Employed	3 (4.5)	98 (8.3)	
Unemployed	63 (95.5)	1083 (91.7)	

^a^Adolescents who did not come back at follow-ups.

## Discussion

This study is one of the first efforts to investigate the long-term effectiveness of a pragmatic multi-setting lifestyle intervention on LTPA levels in boys and girls in the early and late stages of adolescence. Our results indicate considerable differences in energy expenditure in LTPA between boys and girls throughout the study. It is also evident that LTPA and related energy expenditure in boys who underwent the intervention since early adolescence showed an increasing trend until young adulthood. Findings emphasized the vital role of initiation time of intervention to improve LTPA levels over time in this age group. However, similar results were not observed in older boys and adolescent girls.

The current study showed that adolescent boys were more active than girls before and even after intervention. These findings confirm previous reports regarding gender-specific patterns of LTPA in both developed and developing countries [2, 10, 29–31]. Various socio-cultural and bio-psychological factors may justify this difference. Studies from Western countries suggested that gender differences in physical activity levels are caused by differences in sports club enrollment rates between boys and girls, lower social support to engage in PA, and less perceived enjoyment for taking part in physical education in girls [10, 32]. More evidence emphasizes a lack of safety perceptions and stress following multiple task expectations and pressures on girls that may lead to lower physical activity [33, 34]. Also, girls’ biological features and sexual maturity at an earlier chronological age lead to reduced physical activity [35]. In Eastern countries, due to traditionalism, social and cultural constraints, and inadequate sports facilities, women’s physical activity participation is far lower than men’s [36]. Studies targeting Iranian populations revealed low self-efficacy, hard-to-find safe and easy-access sports facilities, and unsupportive families as the main barriers for women that may cause gender differences in physical activity levels [30, 37, 38]. The mentioned factors can be the main reasons for different results in adolescent boys and girls after the current intervention.

Our results revealed that the current healthy lifestyle intervention improved LTPA levels in younger boys rather than their older counterparts. Findings emphasize the vital role of intervention onset time to achieve expected goals in this group. Previous studies have shown controversial results in this regard. Slujis et al. stated that lifestyle interventions in older adolescents were more successful due to more possibility for changes in this group than in younger ones [31]. However, other studies have shown more success in the latter group due to the longer attendance at school, resulting in more prolonged exposure to the intervention [39, 40]. Following graduation from high school, the intervention population was no longer exposed to the school-based arm of the intervention, so the optimal results regarding LTPA may not be observed [41]. In Iran, physical activity decreases across the lifespan, similar to other countries, especially in the transition from secondary to high school [42, 43]. Most Iranian high school students start preparing for university entrance exams; thus, they spend less time on physical activity and most of their time studying. These factors are more salient for Iranian girls due to parental overprotection and restriction about staying out of the house [37, 44]. Previous studies have also proven the impact of university entrance exams and prioritizing studying over physical activity in Taiwanese, Australian, and English girls and Vietnamese adolescents [42, 45, 46]. Therefore, the current results regarding the failure of the intervention to improve LTPA in the last years of adolescence can be sought in the education system and educational expectations of adolescents in this age group and their families.

The current study was one of the first trials in the Eastern-Mediterranean region, evaluating the long-term changes in energy expenditure in LTPA in adolescent boys and girls who have undergone a practical, healthy lifestyle intervention from early or late adolescence. The results of the present study not only provide the possibility of examining the process of LTPA during a long-term pragmatic lifestyle intervention in both sexes and provide valuable information about the effect of the onset time of these interventions in adolescents. However, the current study had some limitations, one of which is the non-randomized design of the study. As a field trial, the TLGS was performed in a middle-class homogeneous urban area, so it was reasonable to be hypothesized that there was no difference among the population covered by the three health care centers in terms of environmental and socio-economic factors that could affect the outcome of the intervention. The assumption was mainly confirmed by comparing the baseline characteristics of the participants in the intervention and control groups except for a limited number of family factors that were more favorable in the control areas. Collecting behavioral information using questionnaire-based methods may increase recall bias. Self-reported assessment of LTPA may not be precise enough to evaluate physical activity levels compared to objective methods [47]. Moreover physical activity was measured using two different questionnaires at baseline (LRC) and follow-ups (MAQ). The current intervention had no fidelity assessment protocol for the participants’ classes. However, since the central part of lifestyle education was conducted in schools where students need to participate every day, the consistency in the delivery of education and other environmental activities was monitored by the school staff and researchers in charge. Moreover, as a part of TLGS, the current study was conducted in urban areas; therefore, the results may not be generalized to suburban and rural populations.

## Conclusion

The current study showed the positive effect of a multi-setting pragmatic intervention on LTPA levels and related energy expenditure in early adolescent boys but not in older boys or adolescent girls. These findings would be valuable to plan tailored interventions to improve physical activity and community health.

## Data Availability

The datasets used and/or analyzed during the current study are available from the corresponding author on reasonable request.

## Abbreviations

LTPA: Leisure-time physical activity
TLGS: Tehran Lipid and Glucose Study
MAQ: Modifiable Activity Questionnaire
SuRFNCD: National Surveillance of Risk Factors of Non-Communicable Diseases
IRAN-ECHO: IRAN-Ending Childhood Obesity
NCD: Non-communicable diseases
METs: Metabolic equivalents
LRC: Lipid Research Clinics
GEE: Generalized Estimating Equations
